# Transcriptional regulator NtrC modulates nitrogen assimilation, virulence, and the extracellular glutamine synthetase activity in *Acinetobacter baumannii*

**DOI:** 10.1371/journal.pone.0341569

**Published:** 2026-01-23

**Authors:** Akram J. Alahmar, Noha M. Elhosseiny, Rehab R. Mahmoud, Ahmed S. Attia

**Affiliations:** 1 Graduate Studies Program, Faculty of Pharmacy, Cairo University, Cairo, Egypt; 2 Department of Microbiology and Immunology, Faculty of Pharmacy, Cairo University, Cairo, Egypt; 3 Department of Pharmacology, Toxicology and Supporting Science, College of Pharmacy, Al-Farahidi University, Baghdad, Iraq; University of Buea, CAMEROON

## Abstract

*Acinetobacter baumannii* is a growing threat characterized by worrisome antibiotic resistance. A deeper understanding of its resistance and virulence mechanisms is essential for developing new and effective treatments. Herein, we explore the role of the two-component (NtrB-NtrC) signal transduction system and two distinct glutamine synthetases (GlnA-1 and GlnA-2) in the nitrogen assimilation, stress response, and virulence in *A. baumannii*. Under nitrogen-limited conditions, the *ntrC* mutant showed significantly defective growth kinetics when ammonium was the sole source of nitrogen, whereas the *glnA2* mutant exhibited an obvious growth defect when putrescine was the sole source of nitrogen. Moreover, under nitrogen limitation, the *glnA1* and *glnA2* expression increased by approximately twofold and ninefold, respectively. An enzymatic activity assay demonstrated that the *A. baumannii* extracellular glutamine synthetase activity is dependent on the type II secretion system (T2SS), confirming our previous results from a T2SS secretome study. Interestingly, this activity is also regulated by NtrC. An infection model using *Galleria mellonella* revealed that the *ntrC* mutant was significantly less virulent than both the wild-type and *glnA2* mutant strains. These results provide new insights into the nitrogen regulatory network and its contribution to the *A. baumannii* virulence, underscoring NtrC as a promising target for future antimicrobial strategies.

## Introduction

*Acinetobacter baumannii* is a highly adaptable nosocomial pathogen equipped with sophisticated virulence factors and defense mechanisms that enable immune evasion and resistance to major antibiotic classes [1]. Its exceptional genetic plasticity further enhances its ability to withstand physiological stress, colonize host niches, and exacerbate infections [2].

Understanding how this microbe utilizes its virulence factors to establish an infection is crucial for developing novel antimicrobials with unconventional mechanisms of action that are less likely to lead to the development of resistance. Extracellular substrates secreted through multiple secretion systems have been shown to significantly enrich the arsenal of the pathogen with diverse virulence factors [3]. Among these systems, the type II secretion system (T2SS) stands out as particularly important. Our group and others have previously characterized the T2SS in *A. baumannii* as a membrane-based machinery that secretes numerous virulence factors, which actively contribute to pathogenesis [4,5].

Our previous analysis of the T2SS secretome *in A. baumannii* AB5075 identified a number of proteins that are involved in the pathway for nitrogen assimilation, such as glutamate dehydrogenase (GdhA) and glutamine synthetase, type I (GlnA-1) [6]. Glutamine synthetase is a vital enzyme in nitrogen assimilation that catalyzes the synthesis of glutamine by transferring an amino group (NH_2_) to glutamate. This activity is usually under the regulation of the nitrogen transcriptional regulatory proteins B and C (NtrB/NtrC) two-component system (TCS) that plays a crucial role in sensing cellular nitrogen levels [7].

Glutamine synthetase (GS) family proteins exhibit significant diversity across various species. Three homologous groups of enzymes belonging to this family (GlnA-1, GlnA-2, and GlnA-3) have so far been described in various species, with GlnA-1 being the most thoroughly studied [8,9]. In the *A. baumannii* AB5075 genome, two distinct genes, *ABUW_1207* (*glnA1*) and *ABUW_1586* (*glnA2*), encode GlnA-1 and GlnA-2, respectively. Although both enzymes share the conserved catalytic domain typical of glutamine synthetases, they may also serve additional functions [10]. Notably, GlnA-2, which was not detected in our T2SS secretome analysis [6], is predicted to be a glutamate-putrescine ligase/transferase, suggesting a possible role beyond conventional nitrogen assimilation.

Polyamines such as putrescine, spermidine, cadaverine, and spermine can be toxic to the cell. Therefore, their catabolism not only enables detoxification of high polyamine concentrations but also provides an alternative source of nitrogen under limiting conditions [11]. The degradation of polyamines, particularly putrescine, as the sole nitrogen source in media has been well characterized in many microorganisms [12]. Putrescine utilization begins with its uptake through a dedicated multi-protein transporter system, enabling intracellular catabolism [8]. Once imported, putrescine is primarily degraded through the primary glutamylation pathway (Puu), which is initiated by a gamma-glutamyl putrescine synthetase (PuuA), resulting in the production of gamma-aminobutyric acid (GABA). A secondary, partially redundant transaminase pathway (Pat/YgjG) converts putrescine into succinate. Both pathways funnel their end products into the tricarboxylic acid (TCA) cycle and can functionally compensate for each other. [13,14].The internal concentration of glutamine is the primary intracellular signal for regulating nitrogen availability in bacteria [15,16]. Nitrogen is essential for the biosynthesis of macromolecules; accordingly, the adaptive response to metabolic stress induced by nitrogen starvation could affect bacterial physiology, including the susceptibility to antimicrobials [17]. The metabolic roles of GS, its regulation through NtrC, and its contribution to virulence have been well-documented [16,18]. However, the extracellular activity and secretion of GS are less frequently described.

Herein, we confirm that *A. baumannii* secreted GS activity is T2SS-dependent and relate this activity to the expression levels of the transcriptional regulator NtrC in both nitrogen-rich and nitrogen-limited conditions. We also demonstrate the significant contribution of the components of the nitrogen assimilation pathway to the virulence of this pathogen.

## Materials and methods

### Bacterial strains and growth conditions

*A. baumannii* strain AB5075 was used as the wild-type (AB5075-WT) [19]. The *gspD* mutant (*gspD*::Tn), which lacks the general secretory pathway protein D (GspD), the T2SS outer membrane gate component, the *ntrC*-mutant (*ntrC::*Tn), and the GlnA-2-mutant (*glnA2::*Tn) were obtained from a transposon mutagenesis library constructed by the Manoil Lab, University of Washington, Seattle [20]. The complemented strain *gspD::*Tn/p*gspD* was constructed as previously described [6]. *A. baumannii* strains and *Escherichia coli* strain DH5α, used as a cloning host, were grown at 37 °C on Tryptic Soy Broth (TSB) with shaking at 180 rpm or TS Agar (TSA). For nitrogen-limited media, *A. baumannii* was grown in liquid M9 minimal salt medium supplemented with defined carbon and nitrogen sources, prepared as previously described [21]. When needed, the media were supplemented with tetracycline to a final concentration of 5 μg/mL and/or apramycin to a final concentration of 25 μg/mL.

### Complementation of the *ntrC::*Tn and *glnA2::*Tn mutants

For the complementation of the *ntrC*::Tn mutant, the primer pair AJ017 (5′-CTT**cccggg**AAGACAGGAACGATTCGCTAATG-3′, the XmaI site underlined), and AJ014 (5′-CCT**ctgcag**TTAAGCTTCTGACAATGT-3′, the PstI restriction site underlined) was used to amplify a 2,604 bp fragment corresponding to the entire *ntrBC* operon, along with 20 bp of the upstream region to include the ribosomal binding site (RBS). For the complementation of the *glnA2::*Tn mutant, the primer pair AN227 (5′-CTT**cccggg**CCAAGGTTTTTTATAATGAG-3′, the XmaI site underlined), and AN228 (5′- CCT**ctgcag**TCAAATATTCCAATGTTGTT-3′, the PstI restriction site underlined) was used to amplify a 1,425 bp fragment corresponding to the open reading frame (ORF) of the entire *glnA2* gene, along with 15 bp of the upstream region to include the RBS. The amplified fragments were subsequently digested with XmaI and PstI (NEB), then ligated into plasmid pMJG120 [22], digested with the same restriction enzymes.

The resultant plasmids (pMJG120-*ntrB*C and pMJG120-*glnA2*) were extracted from positive transformants, verified through DNA sequencing, and subsequently introduced into electrocompetent *ntrC::*Tn and *glnA2::*Tn cells. This resulted in the generation of the complemented strains *ntrC::*Tn/p*ntrBC* and *glnA2::*Tn/p*glnA2*.

### Bioinformatics analysis

The putative glutamine synthetase (GlnA-2) in *A. baumannii* was analyzed in silico to clarify its annotation and potential function. Protein sequences of GlnA-1 and GlnA-2 were retrieved from the NCBI database (protein ID AKA30958.1 and AKA31324.1, respectively). Clustal Omega was used to perform a pairwise sequence alignment between the *A. baumannii* GlnA-1 and GlnA-2 to determine the percent similarity and identity. Blastp (https://blast.ncbi.nlm.nih.gov/Blast.cgi) was used to check the degree of conservation of each of GlnA-1 and GlnA-2 by performing a blast against *Acinetobacter* taxid (taxid 469), as well as other bacterial genera, by excluding *Acinetobacter* from the screened database. Conserved domains, sites, and residues were analyzed using InterPro (https://www.ebi.ac.uk/interpro/) to explore potential functional differences between the two enzymes based on their amino acid sequences. The recently solved crystal structure of the *A. baumannii* GlnA-1 was retrieved from the RCSB Protein Data Bank (https://www.rcsb.org/) (PDB ID. 9K2N), and the structure of GlnA-2 was predicted using the AlphaFold2 online webserver of Neurosnap (https://neurosnap.ai/service/AlphaFold2) using the default settings and without imposing a template protein [23]. The two structures were superimposed for comparison using UCSF ChimeraX (version 1.7) [24], and they were color-edited to highlight specific domains in both proteins.

### Growth kinetics in nitrogen-rich and nitrogen-limited media

Overnight cultures of the WT, *ntrC::*Tn, *glnA2::*Tn, *glnA2::*Tn/p*glnA2*, and *ntrC::*Tn/p*ntrBC* in TSB were prepared and used as an inoculum. For growth curves in rich medium (TSB), cultures were adjusted to an OD_600_ of ≈ 1.0 using TSB, then diluted 1:100 and incubated at 37 °C with shaking at 180 rpm. The OD_600_ of the cultures was measured at regular intervals of one hour from 0 to 8 h post-inoculation.

For standard M9 minimal medium (M9-NH_4_), three formulations containing increasing concentrations of ammonium chloride [0.0% (ammonium-free), 0.1% w/v, and 0.2% w/v], which represent the sole nitrogen source, were prepared and used separately. Regarding the modified M9 minimal medium (M9-Put), which was used specifically to test the function of GlnA-2, ammonium chloride was replaced with putrescine dihydrochloride (1,4-diaminobutane dihydrochloride) at a final concentration of 2 mg/mL(0.2% w/v) as the sole nitrogen source [8,10]. Pellets from TSB overnight cultures were washed three times with M9 medium, then resuspended in minimal media to an OD600 of ≈ 1.0. Then, the cultures were diluted 1:50 in M9 and incubated at 37 °C with shaking at 180 rpm. Under different growth studied conditions, the OD_600_ of all cultures was monitored for 24 h post-inoculation,.exept for the *ntrC::*Tn mutant grown in standard M9 minimal medium (NH_4_Cl, 0.1% w/v), which was monitored for 30 h post-inoculation. The recorded readings were used to generate growth curves by plotting OD_600_ against time.

### Glutamine synthetase activity assay

Cell-free culture supernatants of the WT, *ntrC::*Tn, *gspD::*Tn, *glnA2::*Tn, *ntrC::*Tn/p*ntrBC*, and *gspD::*Tn/p*gspD* were prepared by spinning down overnight cultures followed by filtering the supernatants through 0.22-μm cellulose-acetate syringe filters (Corning). The cell-free supernatants were concentrated 100 × using polyethersulfone column concentrators (MWCO 10 kDa; GE Healthcare). For normalization, the total protein content was quantified using the Pierce Bradford Protein Assay kit (Thermo Fisher Scientific). The GS activity in the normalized cell-free culture supernatants was measured using the (GS) Activity Colometric Assay Kit (Abcam). The absorbance of the ADP-colorimetric product was monitored at OD_570_ in a kinetic assay over 3 hours, and GS activity was determined calorimetrically in nmol/min/mg, according to the manufacturer’s instructions.

### RNA isolation, and real-time reverse transcriptase polymerase chain reaction (RT-RT-PCR)

WT was grown in both TSB and standard M9 medium (NH₄Cl, 0.1% w/v) to mid-logarithmic phase (TSB OD_600_ ≈ 0.4, M9 OD_600_ ≈ 0.6–0.7). Cells were harvested by centrifugation at 2,600 × *g* for 5 min at 4 °C. RNA was extracted using the GeneJET RNA Purification Kit (Thermo Fisher Scientific) according to the manufacturer’s instructions. One μg RNA was then treated with the DNase I, RNase-free enzyme (Thermo Fisher Scientific) to get rid of any possible DNA carryover, then the RNA was used as a template for cDNA synthesis using the RevertAid First Strand cDNA Synthesis Kit (Thermo Fisher Scientific) according to the manufacturer’s instructions and employing random hexamer primers.

Real-time RT-PCR was performed using the Maxima SYBR Green PCR Master Mix (2×) (Thermo Fisher Scientific) in a Rotor-Gene Q thermocycler (Qiagen) for the expression analysis of the genes: *ntrC*, *glnA1*, and *glnA*2. The *16S rRNA* was used as a housekeeping gene for normalization. The primer pairs used are listed in S1 Table in the Supplementary Data. To analyze the data sets, Excel (Microsoft) was used to calculate fold changes, applying the ^∆∆^Ct relative quantification method following normalization by the *16S rRNA* message amplification.

### *Galleria mellonella* killing assay

To assess the contribution of different genes to *A. baumannii* pathogenicity, a *G. mellonela* killing assay was performed. The assay was repeated three independent times using larvae at the fifth instar stage (n = 5, 7, and 15/per group). The hemocoel of each larva was injected with 10 μL of bacterial suspension containing an infection dose of 1–2 × 10^5^ colony-forming units (CFUs). If the calculated CFU count deviated from the target range, the experiment was discarded. The larvae were incubated under controlled conditions in complete darkness at 37°C. The survival rates of the infected larvae were systematically monitored over a five-day period to assess the potential pathogenicity of each bacterial strain tested [4].

### Statistical analyses

Statistical analyses were conducted using GraphPad Prism software (version 8.01) (GraphPad Software, Inc., USA). For comparisons involving three or more groups, variance was assumed to be homogeneous, and significance was determined using one-way analysis of variance (ANOVA), followed by the Newman–Keuls multiple comparison test. Differences were considered statistically significant at (*p ≤ 0.05) and (**p ≤ 0.01), with 95% confidence intervals. In the case of real-time data of the fold-increase in transcriptional level is presented, using the normalized level of transcripts in rich media as a calibrator. Survival data from larval killing assays were analyzed using the log-rank (Mantel-Cox) test to assess differences in survival rates among experimental groups. Differences at (*p > 0.05) were considered statistically significant, and survival curves were generated with 95% confidence intervals.

## Results

### NtrC and GlnA-2 are important for the normal growth of *A. baumannii* under different nitrogen-limited conditions

**Fig 1:**
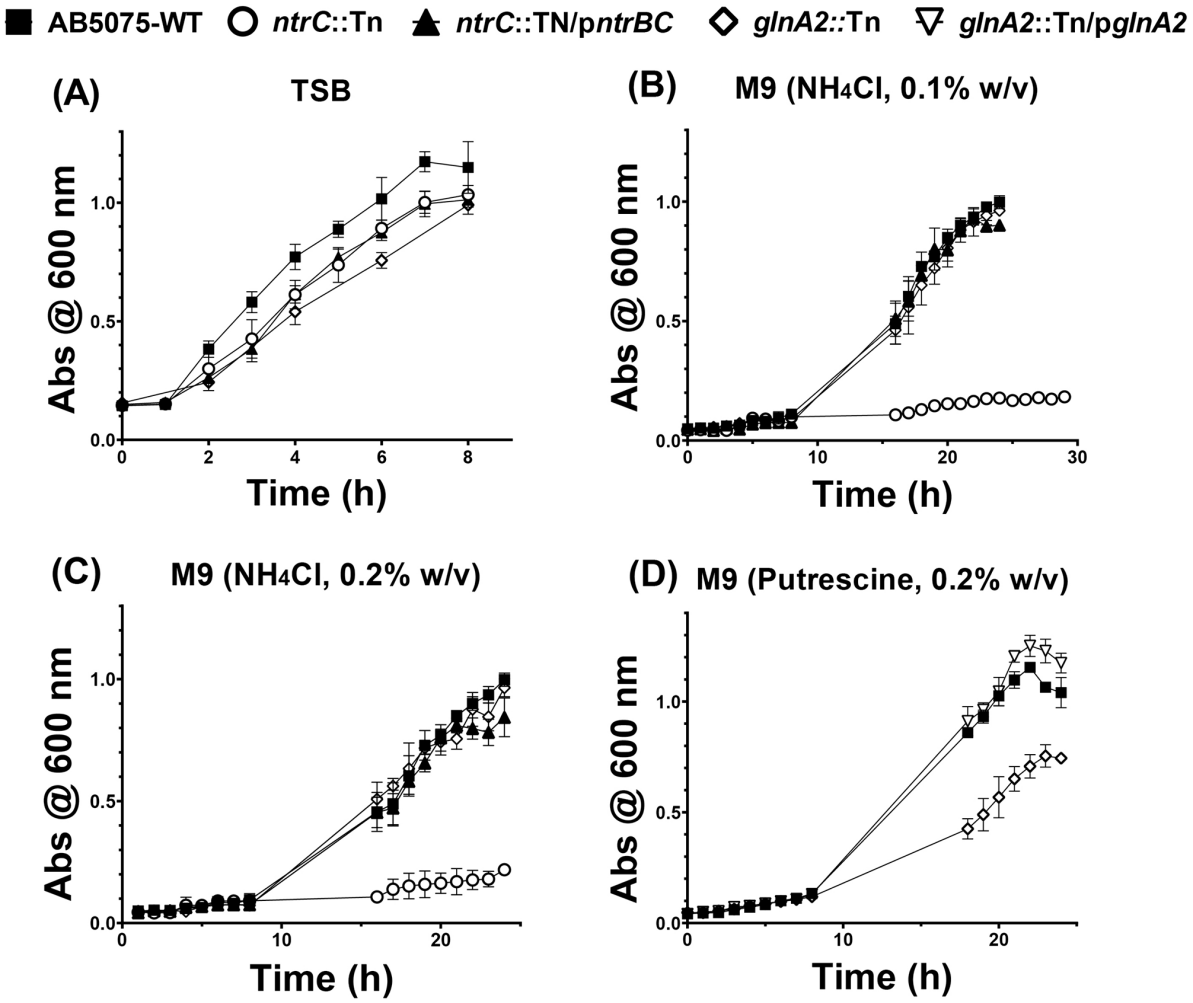
Schematic diagram showing growth dynamics (A) nutrient-rich TSB media, and nutrient-limited M9 minimal media supplemented with a sole nitrogen source as follows: (B) ((M9-NH_4_) is (NH_4_Cl, 0.1% w/v), (C) (M9-NH_4_) is (NH_4_Cl, 0.2% w/v), and (D) (M9-Put) is (Put, 0.2% w/v). Growth curves were generated using GraphPad Prism (version 8). Error bars indicate standard errors of the mean (SEM) of three replicas.

In TSB rich medium, the *ntrC::*Tn mutant did not exhibit any growth defect (Fig 1A). Moreover, the *glnA2::*Tn mutant did not exhibit any growth deficiency in rich or minimal media (M9-NH_4_) (Fig 1A, 1B, and 1C).

In standard M9 minimal medium, which represents the first nitrogen-limited growth conditions in this experiment, none of the tested strains (WT, *ntrC::*Tn, *glnA2::*Tn, and *ntrC::*Tn/p*ntrBC*) exhibited detectable growth when the medium was completely deprived of ammonium (S1 Fig). Upon comparing the growth under the two ammonium concentrations (0.1% w/v and 0.2% w/v), the strains exhibited similar growth patterns, with no marked differences observed between the two conditions (Fig 1B and 1C). Notably, the growth of the *ntrC::Tn* mutant was considerably repressed in the two concentrations of ammonium tested compared to the other strains (Fig 1B and 1C), with a slight improvement in growth profile observed after 24 h in the M9 medium (NH_4_Cl, 0.1% w/v) (Fig 1B). Normal growth was restored in the complemented mutant *ntrC::*Tn/p*ntrBC*, which grew in both ammonium concentrations in a pattern comparable to that of the WT (Fig 1B and 1C).

In the modified M9 minimal medium (putrecsine; 0.2% w/v) the *glnA2::*Tn mutant exhibited a pronounced, yet partial, growth defect (Fig 1D). The defect was restored in the complemented mutant *glnA2::*Tn/p*glnA2* to a pattern comparable to that of the wild-type (Fig 1D)

These findings are consistent with the established role of NtrC in nitrogen assimilation under stress conditions and nitrogen limitation. The result indicates that the NtrB-NtrC two-component system is probably the primary regulator for nitrogen assimilation in *A. baumannii*. Additionally, these findings further suggest that GlnA-2 appears to play an important role in *A. baumannii* growth kinetics when putrescine, rather than ammonium chloride, is the sole nitrogen source.

### GlnA-2 is a predicted gamma-glutamylputrescine synthetase in *A. baumannii* AB5075

GlnA-2 is recognized in the NCBI as a glutamine synthetase family protein; however, unlike GlnA-1, it was not found as a T2SS-secreted protein in our previous secretome analysis [6]. The presence of GlnA-2 in the *A. baumannii* proteome was predicted through BlastP analysis using the GlnA-1 sequence as a query.

A pairwise sequence alignment revealed that the similarity between GlnA-1 and GlnA-2 of *A. baumannii* is high, at 57%, and the Blastp analysis showed that proteins are highly conserved across the *Acinetobacter* taxid (S2 and S3 Figs). The in-silico results also indicated that although the GlnA-1 and the GS family protein (GlnA-2) were found among numerous Gram-negative species (S4 and S5 Figs), GlnA-2 was annotated under a different designation, namely gamma-glutamylpolyamine synthetase or gamma-glutamylputrescine synthetase. This protein shares significant sequence identity with gamma-glutamylputrescine synthetase PuuA from *A. baumannii* WM99c (97.25%), *Klebsiella pneumoniae* (98.1%), *Pseudomonadota* (95.9%), *Acinetobacter pittii* PHEA2 (95.4%) and *E. coli* K-12 (46.6%) (S6 and S7 Figs).

**Fig 2:**
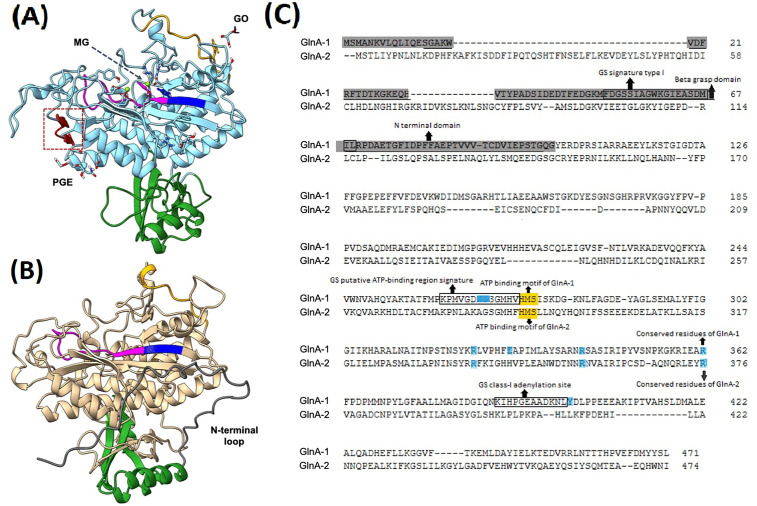
Sequence and structural features of the *A. baumannii* glutamine synthetase family proteins. Schematic diagrams representing (A) the solved crystal structure of the *A. baumannii* GlnA-1 (PDB ID. 9K2N), and (B) the AlphaFold2 predicted structure of the *A. baumannii* GlnA-2. Color highlighting was applied to both structures as follows: the blue and buff regions represent the GS catalytic domain, the N-terminal domain is in green, the ATP-binding signature sequence is in magenta, the ATP-binding motif (HMS) is in blue, and the GS-adenylation site is in orange. Strands β6 and β7 in GlnA-1 are drawn in red and are highlighted with a red dotted box. The long N-terminal loop characteristic of GlnA-2 is colored dark grey. The figures were generated and edited in UCSF ChimeraX version 1.7. **(C)** Amino acid sequence alignment of GlnA-1 and GlnA-2, showing the common and unique sequence features, is highlighted as follows: the ATP-binding motif (HMS) is in yellow, the N-terminal domain of GlnA-1 is in dark grey, the conserved residues are in purple, the beta grasp domain of GlnA-1 is underlined, and the GS signature sequences of GlnA-1 are enclosed within rectangles. The alignment was created using Clustal Omega, and the sequence features were derived from the analysis in InterPro.

Additionally, in silico analysis using InterPro, a tool for protein family classification, indicated that AB5075 GlnA-2 (GO:0006598 for polyamine catabolic process and GO:0006542 for glutamine biosynthetic process) has the glutamine synthetase (GS) catalytic domain, which it shares with GlnA-1. However, it lacks both the N-terminal domain and the GS-beta grasp domain found in typical GlnA-1 (Fig 2). The structure of GlnA-1 from *A. baumannii* has recently been solved by another group and deposited in the PDB (9K2N). Structural prediction of GlnA-2, and its comparison to GlnA-1 showed that GlnA-2 is predicted to have an N-terminal long loop that is not found in GlnA-1, although the general architecture of the N-terminal domain is similar in both proteins (Fig 2A and 2B). Both GlnA-1 and GlnA-2 possess a conserved ATP-binding motif composed of the amino acids His-Met-Ser: residues (274–276) in GlnA-1 and (287–289) in GlnA-2. Moreover, the analysis also showed that GlnA-1 (GO:0006542 for glutamine biosynthetic process and GO:0019740 for nitrogen utilization) contains six conserved residues (Asn267, Arg324, Glu330, Arg342, Arg362, and Tyr400) and three conserved binding sites involved in substrate binding for L-glutamate and ATP (Fig 2C). Conversely, GlnA-2 retains only three conserved arginine residues (Arg339, Arg357, and Arg376) that are specifically associated with L-glutamate binding (Fig 2C). Notably, upon comparing the two structures, it was noted that GlnA-2 lacks two beta-strands (β6 and β7) that are found in the structure of GlnA-1, and thus slightly changes the architecture of the GS domain in GlnA-2 (Fig 2A and 2B). These findings indicate that although GlnA-2 shares structural and domain organization similarities with GlnA-1, it exhibits different features, which could account for its potential role as a poly- and/or mono-amino glutamate ligase enzyme.

### The T2SS mediates the extracellular GS activity of *A. baumannii* in an NtrC-dependent manner

**Fig 3:**
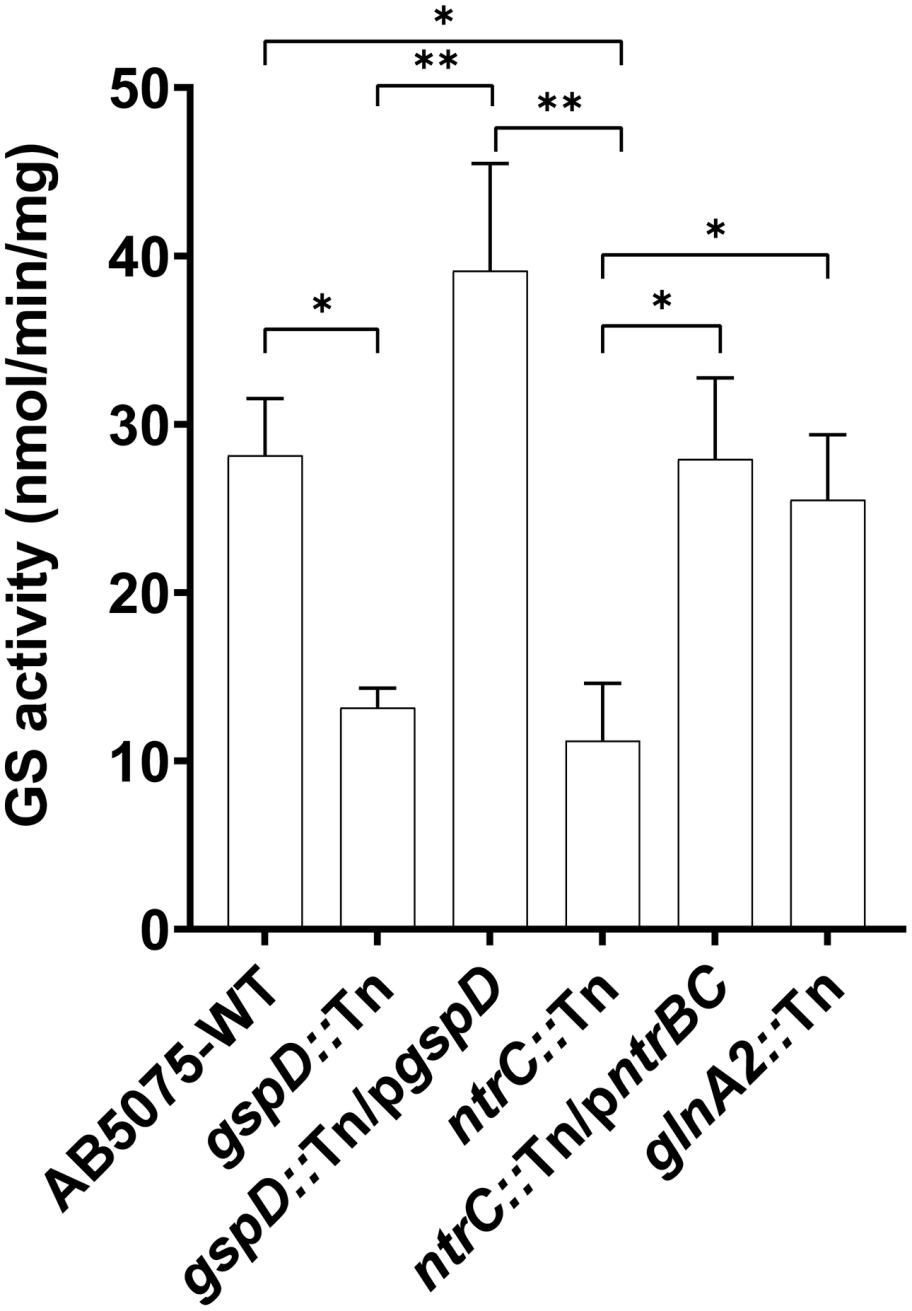
*A. baumannii* secretes glutamine synthetase (GS) in a manner dependent on the type II secretion system (T2SS). A bar graph illustrating the measured extracellular GS activity across various strains. The GS activity was measured at two time points (t₁ = 90 min, t₂ = 180 min), and the mean change in activity between these time points was calculated. Error bars indicate standard errors of the mean (SEM) of two biological replicas.

In our previous study, mass spectrometric findings suggested that GlnA-1 is secreted via T2SS in *A. baumannii* [6]. To validate this hypothesis, GS activity was assessed in cell-free supernatants of the T2SS mutant *gspD::*Tn, and its complemented construct. The GS activities were quantified after normalizing the OD₅₇₀ measurements to total protein content in the culture supernatants (S8 and S9 Figs). The results revealed a significant reduction of about 55% in GS activity in the supernatants of the T2SS mutant compared to that of the WT-AB5075 strain. Notably, extracellular GS activity in the complemented strain *gspD::*Tn/p*gspD* surpassed that of the wild-type levels by approximately 30%, further confirming that GS secretion is dependent on a functional T2SS (Fig 3).

The assay was also performed on the supernatants of the *ntrC::*Tn to assess its regulatory role in modulating secreted GS activity. The results showed a pronounced decrease in the activity of about 65% compared to that of the WT (Fig 3). Moreover, this decrease was fully restored in the complemented NtrC-mutant (*ntrC::*Tn/p*ntrBC*) to the levels seen in the supernatant of the WT (Fig 3). No significant reduction in the extracellular GS activity was observed in the *glnA2::*Tn mutant, the putative glutamate-putrescine ligase mutant. These results indicate that the extracellular GS activity of *A. baumannii* is dependent on the regulatory role of NtrC rather than on GlnA-2. Consistent with its predicted function as a glutamate–putrescine ligase, GlnA-2 does not likely appear to contribute to GS activity; moreover, its absence from the T2SS secretome precludes a role in extracellular GS activity.

### The *ntrC*, *glnA1*, and *glnA2* genes are overexpressed under nitrogen limitation

**Fig 4:**
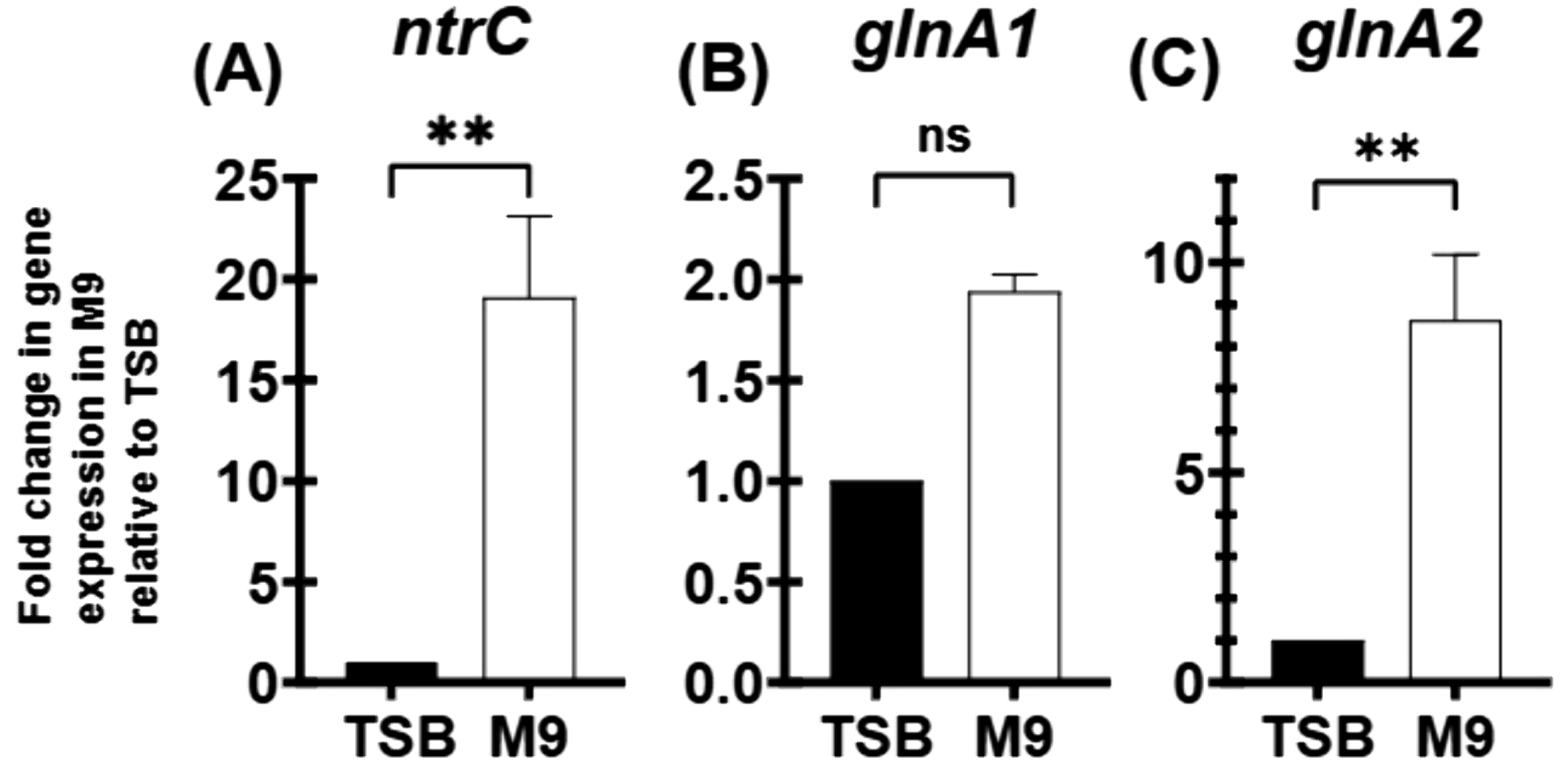
Transcriptional level of nitrogen assimilation genes in M9 relative to TSB. Bar graphs representing the relative expression of nitrogen assimilation genes: (A) *ntrC,* (B) glutamine synthetase, type-I (*glnA1*), and (C) predicted glutamate-putrescine ligase (*glnA2*), in standard M9 relative to TSB. The expression level in the TSB sample was used as a calibrator. Readings represent the mean of four replicas. Error bars indicate the standard error of the means.

A real-time PCR analysis illustrated that a remarkable stress response was observed, as evidenced by a significant upregulation of the *ntrC* gene expression when *A. baumannii* was cultured in the nitrogen-limited M9 medium, exhibiting a 19-fold increase compared to that in the nitrogen-rich condition (Fig 4A). Further analysis demonstrated that this upregulation was accompanied by a moderate 2-fold increase in *glnA1* expression (Fig 4B), whereas *glnA2* displayed a more notable induction, reaching approximately 9-fold increase (Fig 4C). These results indicate that NtrC is required for modulating the stress response induced by nitrogen limitation. They also indicate that both glutamine synthetase family proteins (GlnA-1 and GlnA-2) are upregulated in response to nutritional limitation, but to varying degrees.

The consistency of these results was validated by analyzing the melting curves, which verified the specificity and efficiency of the primers (S10 Fig).

### The nitrogen assimilation pathway is essential for the *A. baumannii* virulence in the *G. mellonella* model

The pathogenicity of the tested strains was investigated using the invertebrate *G. mellonella* infection model. Significant differences in survival rates were observed among the strains at a 95% confidence interval, supporting the reliability of the findings (S11 Fig).

**Fig 5:**
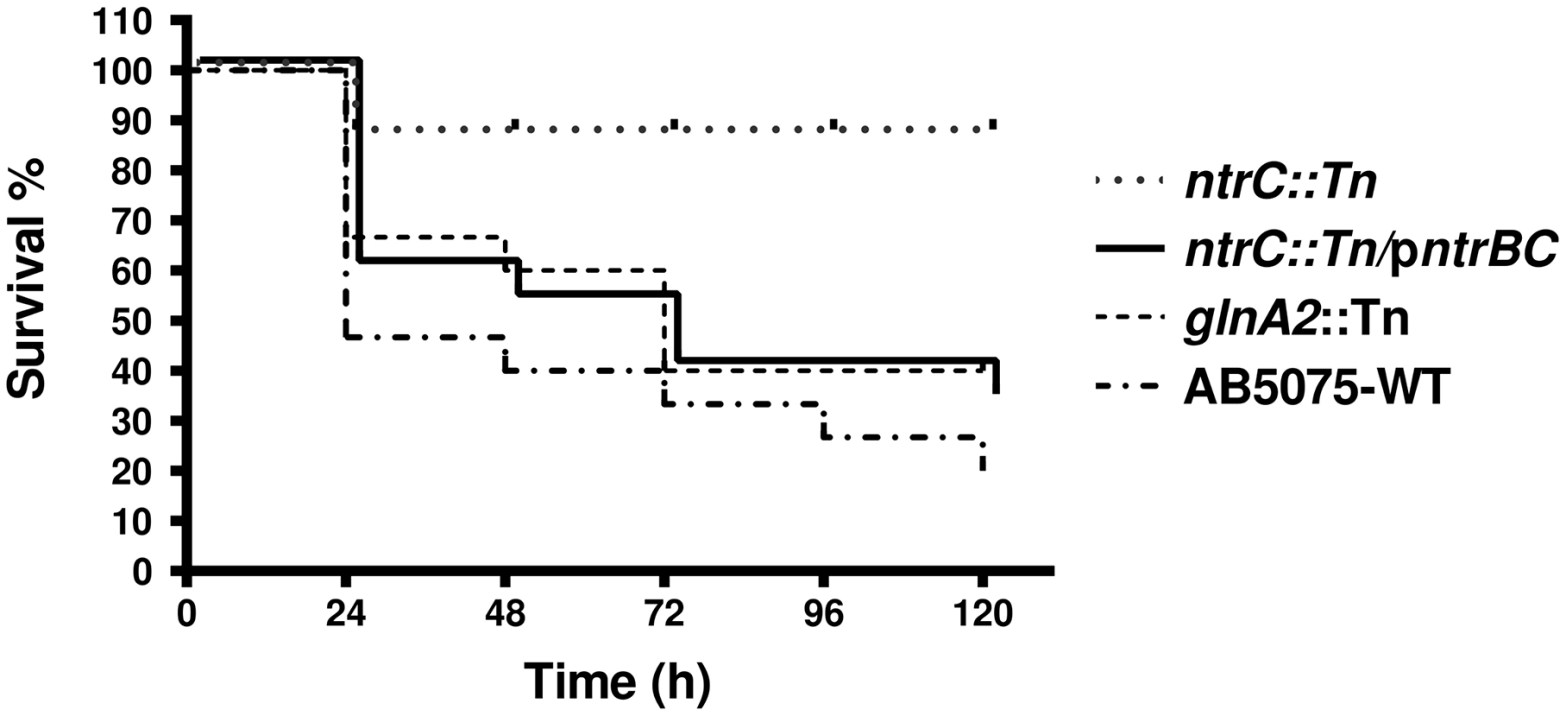
Contribution of nitrogen assimilation proteins in the virulence of *A. baumannii.* A Kaplan-Meier plot representing survival analysis of *G. mellonella* larvae infected with *A. baumannii* strains over time (5 days = 120 hours). Four groups of larvae were infected in three independent biological replicates of either WT, *glnA2::*Tn, *ntrC::*Tn, or *ntrC::*Tn/p*ntrBC*. The survival rates were recorded over time and analyzed statistically. The survival rates for all groups were significantly different, with a *p*-value of 0.001.

The WT strain exhibited the highest virulence among all tested strains, causing an 82% mortality rate with the majority of mortality observed within the first 24 h of infection (Fig 5). Interestingly, the *ntrC::*Tn mutant exhibited a significant attenuation of virulence, with only a 15% larval mortality rate observed over the five-day monitoring period (Fig 5). The pathogenicity was partially restored in the complemented *ntrC* mutant compared with the WT, as it displayed a significant mortality rate of 65%, albeit with a delayed effect, reaching this threshold after 120 h post-infection. Surprisingly, the *glnA2::*Tn mutant maintained a notable lethality, resulting in 64% larval mortality within 72 h (Fig 5). These findings underscore the crucial role of the nitrogen assimilation pathway components, particularly NtrC, in the virulence of *A. baumannii*.

## Discussion

In this study, our aim was to investigate the relationship between regulators of the nitrogen cycle, specifically the NtrB-NtrC two-component system (TCS), and glutamine synthetase family proteins in *A. baumannii*. We were particularly interested in glutamine synthetases as components of the nitrogen cycle because 1) their expression profile and their relationship with NtrC have not been studied before in *A. baumannii*, 2) one of our previous studies characterizing the T2SS secretome of *A. baumannii* revealed that GlnA-1 could be a potential T2SS substrate [6]. We aimed to confirm the T2SS-dependent GS activity and determine whether this activity could be modulated through NtrC.

First, nitrogen-limited conditions were designed to impose metabolic stress while preserving minimal nutrient requirements for pathogen survival. An ammonium concentration of 0.1% provided a reproducible stress condition for all studied strains, whereas complete ammonium removal abolished the growth. This inhibition was expected, given the pivotal roles of nitrogen in all biological processes [25]. Increasing ammonium concentration to 0.2% did not measurably alter the stress response. Notably, we observed that the *A. baumannii* wild-type growth under nitrogen-limited conditions was enhanced when putrescine was the sole nitrogen source compared to in the presence of ammonium chloride. This may be explained by the fact that, at the concentrations used in this experiment, putrescine could provide both nitrogen and a supplemental carbon source, since the end products of its metabolism can be funneled into the TCA cycle. Similar observations have been reported in *E. coli* and *Pseudomonas syringae* [12,26–28].

The overall regulatory role of NtrC in the nitrogen stress response was investigated by measuring the growth rate of an NtrC mutant (*ntrC::*Tn) in minimal versus rich media. A substantial growth defect was observed for the *ntrC::*Tn strain under nutrient-limiting conditions. The mutant started to recover after 24 h of growth in this nitrogen-limited condition. These observations suggest that *A. baumannii* may gradually adapt to nutrient-deprived conditions, potentially through the activation of alternative stress response regulatory systems, regardless of whether the medium is rich or minimal. Examples of these alternative regulatory mechanisms are the MerR-family transcription factor TnrA in *Bacillus subtilis* and the CRP-family transcription factor NtcA in cyanobacteria, which promote the expression of genes involved in nitrogen assimilation [25].

Through GS enzymatic assays on cell supernatants, we observed a significant reduction in the GS extracellular activity of the *gspD*::Tn strain, which carries a transposon insertion disrupting the T2SS exit pore. This activity was restored in the complemented strain *gspD*::Tn/p*gspD*, further substantiating the hypothesis that a GS enzyme or more is actively secreted through the T2SS into the extracellular milieu. Moreover, this finding indicates that the observed extracellular GS activity results from active secretion through the T2SS, rather than from potential leakage associated with cell lysis. The extracellular activity of the GS enzyme suggests it may play an important role in helping the pathogen evade the host immune response or in supporting bacterial adaptation and survival. Similar extracellular functions have been observed in other organisms, such as *Mycobacterium tuberculosis*, where the extracellular GS was identified as a potential drug target. Inhibiting GS activity in *M. tuberculosis*, either using the chemical inhibitor L-methionine-SR-sulfoximine (MSO) or by applying antisense oligodeoxyribonucleotides targeting GS mRNA, disrupts cell wall formation [29,30]. Interestingly, nonpathogenic mycobacteria, which do not secrete glutamine synthetase extracellularly at all, showed no growth inhibition when exposed to the GS inhibitors. [30]. This supports the notion that, similar to the gamma-glutamyl transferase observed in *A. baumannii*, variations in the extracellular secretion of glutamine synthetase may be associated with differences in the pathogenicity of clinical isolates [4]. To explore the role of GlnA-1 in *A. baumannii*, either intracellularly or extracellularly, our study aimed to evaluate glutamine synthetase activity in a *glnA1* transposon mutant (*glnA1*::Tn) and correlate these findings with outcomes from an infection model to assess the impact of *glnA1* disruption on virulence. Although this mutant strain was included in the transposon mutant library, we obtained from the Manoil Lab, it was unfortunately not recoverable. This suggests that *glnA1* may be essential for viability in the AB5075 background under our storage or growth conditions. Future work could include directed mutagenesis of this gene to allow further characterization of the GlnA-1 protein in *A. baumannii*.

Notably, the GS-family protein GlnA-2 in *A. baumannii* does not contribute to the extracellular GS activity. This observation implies that GlnA-1 is likely the major contributor to this activity, being specifically secreted in a T2-dependent manner. This observation also suggests that GlnA‑2 is either not a glutamine synthetase or that its activity is limited to the intracellular environment. Furthermore, the deletion of *ntrC* resulted in a substantial decline in extracellular GS activity.

The GlnA-1, encoded by *ABUW-1207*, and GlnA-2, encoded by *ABUW-1586,* are annotated as members of the glutamine synthetase family proteins in *A. baumannii.* Although our analysis revealed that these proteins are similar in sequence and structure, we identified substitutions in essential residues within the active site of GlnA-2 compared to GlnA-1, suggesting that GlnA-2 in *A. baumannii* may not possess glutamine synthetase activity. Previous studies have reported that the GlnA, type II enzymes either had exclusive gamma-glutamylputrescine synthetase activity, such as in the archaea *Haloferax mediterranei* [31], or showed a dual glutamate-ammonia/glutamate-polyamine ligase activity, such as PuuA of *Streptomyces coelicolor,* and experimentally in *E. coli* K-12 [10,11].

The growth curve analysis revealed that the *glnA2::*Tn mutant did not exhibit a growth defect in nitrogen limitation (M9-NH_4_). As previously mentioned, our standard M9 medium was not completely devoid of ammonium salt. This implies that in the presence of this minimal amount of nitrogen in the medium, GlnA-1 contributed the predominant portion of required GS activity, compared to GlnA-2. In contrast, when putrescine was provided as the sole nitrogen source, the *glnA2::Tn* mutant exhibited a pronounced but incomplete growth defect. A similar phenotype has been reported following disruption of the gamma-glutamylputrescine synthetase (PuuA) in *E. coli* [10]. The partial growth observed in the *glnA2-*mutant under putrescine conditions likely reflects the compensation through an intact transaminase backup pathway, which is known to be induced under nitrogen-limited conditions (M9-Put) [13]. Restoration of growth in the complemented mutant *glnA2::Tn*/p*glnA2* further supports the concept of the compensatory function between these pathways. This scenario is very likely, since previous papers reported that inhibiting the entire putrescine catabolism needed the two pathways to be blocked together [13]. These distinct growth patterns in presence of different nitrogen sources are consistent with the functional prediction of GlnA-2 as a gamma-glutamylputrescine synthetase rather than as a conventional glutamine synthetase.

Interestingly, the AB5075 genome encodes multiple putrescine transporters as well as genes belonging to both putrescine catabolic pathways, including a putrescine importer encoded by *ABUW_0857*, aldehyde dehydrogenase encoded by *ABUW_3390*, 4-aminobutyrate transaminase encoded by *ABUW_0203* (*gabT*), succinate-semialdehyde dehydrogenase encoded by *ABUW_0204* (*gabD1*), and betaine aldehyde dehydrogenase encoded by *ABUW_2808* (*ydcW*)*.* These genes have already been characterized as components of polyamine/putrescine utilization pathways in many other Gram-negative bacteria, such as *E. coli* and *P. aeruginosa* [8,12,14,32]. Notably, the latter two enzymes are major components of the transaminase pathway in these pathogens [33,34]. The presence of these genes, together with our functional data on *glnA2*, supports the possibility of metabolic flexibility in *A. baumannii* for utilizing polyamines as alternative nitrogen sources, a trait that may contribute to its persistence across diverse environmental and host-associated niches [35].

NtrC, the transcriptional regulator, regulates the expression and activity of GS in many species [18,36–39]. The regulatory function of NtrC in *A. baumannii* has been highlighted in a previous study, and the transcriptomic analysis identified 253 genes with differential expression patterns, including 80 genes that exhibited at least a two-fold upregulation and 173 genes that were significantly down-regulated in the Δ*ntrC* strain [40]. Interestingly, the GS-coding genes were not identified in this transcriptomic analysis. Most probably, this is because the authors used a rich medium for growth, rather than minimal media.

Building upon these findings, we compared the transcription of *ntrC*, *glnA1*, and *glnA2* in nutrient-rich versus minimal media. A notable induction of all three genes was observed under nutrient-limited conditions, with *ntrC* demonstrating the most pronounced upregulation. Conversely, GS-coding genes exhibited varying levels of upregulation. The expression of *glnA1* exhibited only a modest twofold increase. We believe that the presence of small amounts of ammonium in the minimal medium was enough to maintain an acceptable level of glutamine synthesis under these conditions, thereby limiting the need for strong transcriptional activation of *glnA1*. In contrast, the *glnA2* gene encoding GlnA-2 displayed significantly higher expression levels. A similar phenomenon has been observed in *S. coelicolor*, where *glnA2* exhibited significantly increased expression under starvation conditions with low glucose and ammonium concentration [11]. It was previously reported that nitrogen starvation induces an increase in the levels of polyamines inside the cell, mainly putrescine [41]. This surge in polyamines following nitrogen limitation may also account for the pronounced overexpression of the *glnA2* gene. This is an expected adaptation to stress response, either as a trial to acquire nitrogen from alternative sources (polyamines), or to maintain cellular homeostasis by tightly regulating polyamine levels inside the cell [12].

The *G. mellonella* killing assay demonstrated that the predicted GlnA-2 of *A. baumannii* contributed modestly to virulence under the tested conditions. GlnA-2 has been reported in other pathogens, such as *M. bovis*, to contribute to pathogenicity [42]. This makes sense since polyamines significantly contribute to virulence, such as modulating the host’s immune defenses, inducing apoptosis, and enhancing biofilm formation [43]. To understand the function of GlnA-2 in *A. baumannii’s* pathogenesis, further investigation is necessary and is an intriguing topic for future research by our team. The killing assay confirmed the role of NtrC as a pivotal virulence determinant, evident by the significant loss of virulence in the *ntrC* mutant. This is consistent with previous investigations in other bacterial species, such as *P. aeruginosa* and *Acidovorax citrulli*, which demonstrated species-specific pathogenic roles of NtrC [37,44].

Overall, we presented one of the first studies to investigate members of the regulatory network of nitrogen assimilation in *A. baumannii*. The remarkable impact of NtrC depletion on bacterial fitness and virulence indicates that targeting NtrC could serve as an effective therapeutic strategy. Our study highlights the importance of further investigating the substrates of T2SS, their contribution to virulence, and how they may be regulated. We also reinforced our previous findings about the T2-dependent secretion of the extracellular glutamine synthetase in *A. baumannii*, a phenomenon rarely reported in bacterial species. Furthermore, we proposed *A. baumannii* uses its putative flexible capacity to utilize nitrogen from diverse sources as an adaptation to stress conditions

## Supporting information

S1 Fig Schematic diagram growth of dynamics under nitrogen-free conditions in M9 minimal medium. (TIF)

S2 Fig A graphic representation of the Blastp analysis of the amino acid sequence of the type-I glutamine synthetase (GlnA-1) of *A. baumannii* AB5075 across *Acinetobacter* taxid (taxid:469). The red lines indicate the high degree of conservation against the entire taxid. The Blastp analysis was performed using the default settings.
(TIF)

S3 Fig A graphic representation of the Blastp analysis of the amino acid sequence of the predicted gamma-glutamyl putrescine synthetase (GlnA-2) of *A. baumannii* AB5075 across *Acinetobacter* taxid (taxid:469). The red lines indicate the high degree of conservation against the entire taxid. The Blastp analysis was performed using the default settings.
(TIF)

S4 Fig A graphic representation of the Blastp analysis of the amino acid sequence of the type-I glutamine synthetase (GlnA-1) of *A. baumannii* AB5075 across other bacterial genera, excluding *Acinetobacter* taxid (taxid:469) from the search query. The red lines indicate the high degree of conservation across the different genera. The Blastp analysis was performed using the default settings.
(TIF)

S5 Fig A graphic representation of the Blastp analysis of the amino acid sequence of the predicted gamma-glutamyl putrescine synthetase (GlnA-2) of *A. baumannii* AB5075 across other bacterial genera, excluding *Acinetobacter* taxid (taxid:469) from the search query. The red lines indicate a high degree of conservation across different genera, while incomplete lines indicate a shorter sequence in the search query. The Blastp analysis was performed using the default settings.
(TIF)

S6 Fig A Clustal Omega pairwise alignment. It was performed between GlnA-2 from *A. baumannii* AB5075 and a Gamma-glutamyl-putrescine synthetase (PuuA) from *A. baumannii* WM99c and *A. pittii*, highlighting (*) identical, (:) conserved, and (.) semi-conserved residues.
(TIF)

S7 Fig A Clustal Omega pairwise alignment. It was performed between GlnA-2 from *A. baumannii* AB5075 and a PuuA from various Gram-negative species, highlighting (*) identical, (:) conserved, and (.) semi-conserved residues.
(TIF)

S8 Fig Total protein quantification of concentrated supernatants secreted by different *A. baumannii* strains. (TIF)

S9 Fig Schematic diagram curves illustrating absorbance changes of the ADP colorimetric product in supernatants from different *A. baumannii* strains, using a GS-positive background as a reference. (TIF)

S10 Fig Melting curve analysis of qPCR primers. Curves are obtained from real-time PCR reactions demonstrating the specificity and efficiency of primers used for the genes of interest and the 16S rRNA gene. (A) *16S rRNA*, (B) *glnA1*, (C) *glnA2*, and (D) *ntrC*.
(TIF)

S11 Fig A Kaplan-Meier plot representing survival curves of *Galleria mellonella* larvae infected with *A. baumannii* strains. Dotted lines around each curve represent 95% confidence intervals. Survival differences among groups were analyzed and considered statistically significant (*p* = 0.001).
(TIF)

S1 Table List of oligonucleotide primers used in this study. (DOCX)

S1 File Alahmar et al-2025- PLOS one -Supplementary Data-File#2 (Raw data). (XLSX)
