# Supplementary figures and images for: Transcriptional regulator NtrC modulates nitrogen assimilation, virulence, and the extracellular glutamine synthetase activity in Acinetobacter baumannii
Source: PLoS One. 2026 Jan 23;21(1):e0341569. doi: 10.1371/journal.pone.0341569 (PMC12829801; doi:10.1371/journal.pone.0341569)

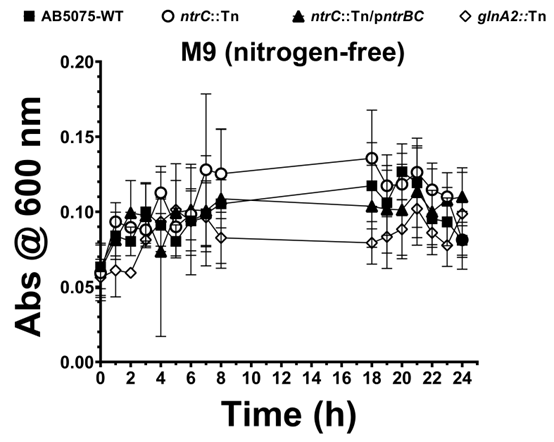

Supplement: S1 Fig — (TIF) [file pone.0341569.s001.tif]

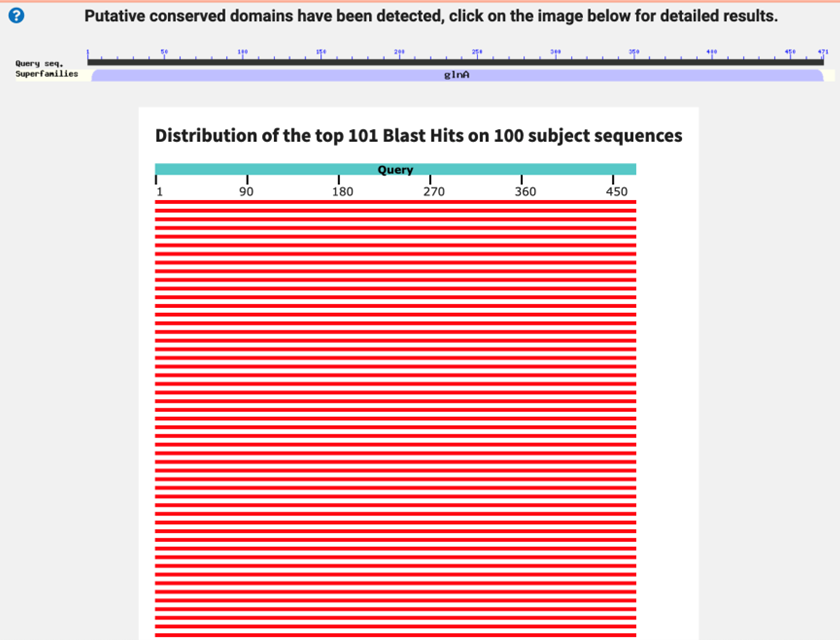

Supplement: S2 Fig — The red lines indicate the high degree of conservation against the entire taxid. The Blastp analysis was performed using the default settings. (TIF) [file pone.0341569.s002.tif]

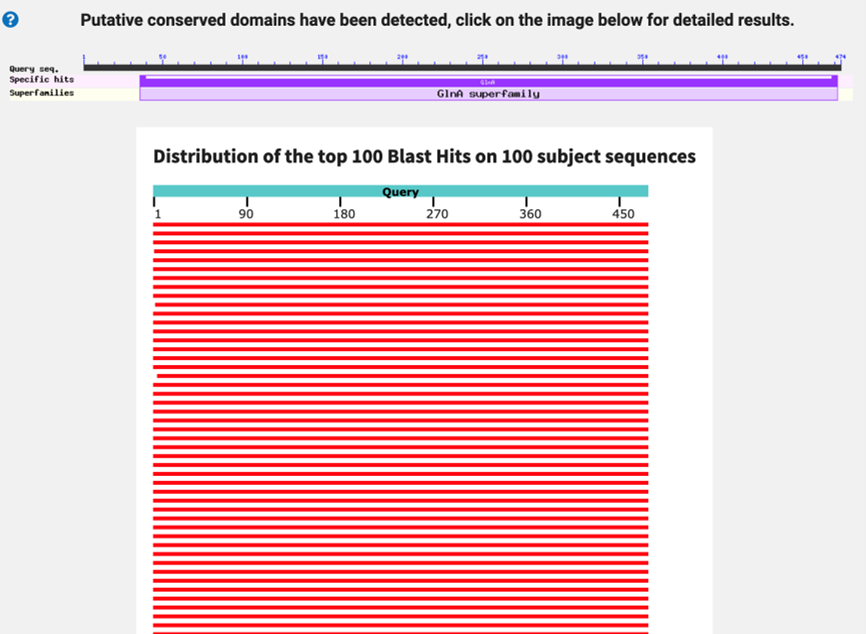

Supplement: S3 Fig — The red lines indicate the high degree of conservation against the entire taxid. The Blastp analysis was performed using the default settings. (TIF) [file pone.0341569.s003.tif]

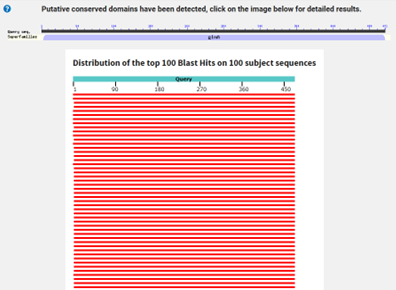

Supplement: S4 Fig — The red lines indicate the high degree of conservation across the different genera. The Blastp analysis was performed using the default settings. (TIF) [file pone.0341569.s004.tif]

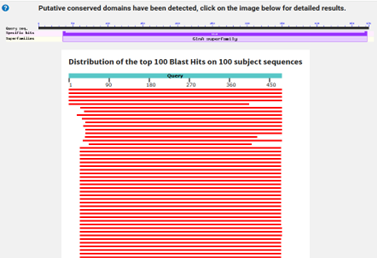

Supplement: S5 Fig — The red lines indicate a high degree of conservation across different genera, while incomplete lines indicate a shorter sequence in the search query. The Blastp analysis was performed using the default settings. (TIF) [file pone.0341569.s005.tif]

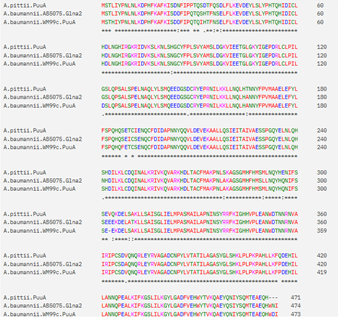

Supplement: S6 Fig — It was performed between GlnA-2 from A. baumannii AB5075 and a Gamma-glutamyl-putrescine synthetase (PuuA) from A. baumannii WM99c and A. pittii, highlighting (*) identical, (:) conserved, and (.) semi-conserved residues. (TIF) [file pone.0341569.s006.tif]

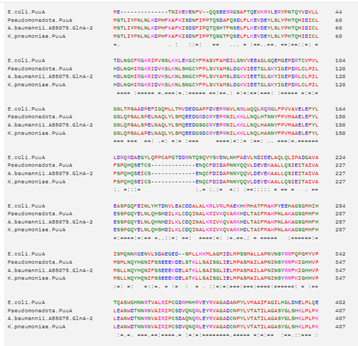

Supplement: S7 Fig — It was performed between GlnA-2 from A. baumannii AB5075 and a PuuA from various Gram-negative species, highlighting (*) identical, (:) conserved, and (.) semi-conserved residues. (TIF) [file pone.0341569.s007.tif]

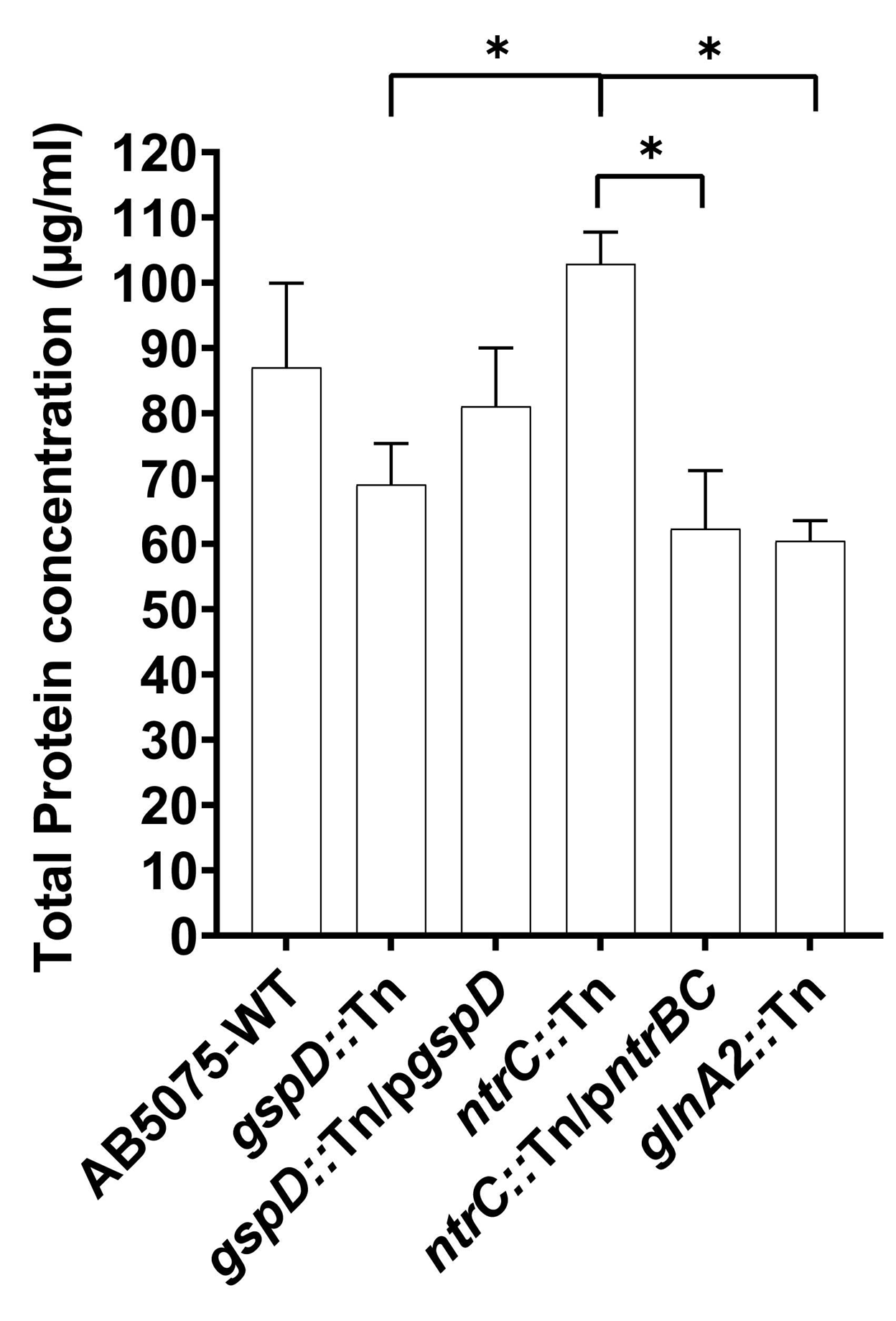

Supplement: S8 Fig — (TIF) [file pone.0341569.s008.tif]

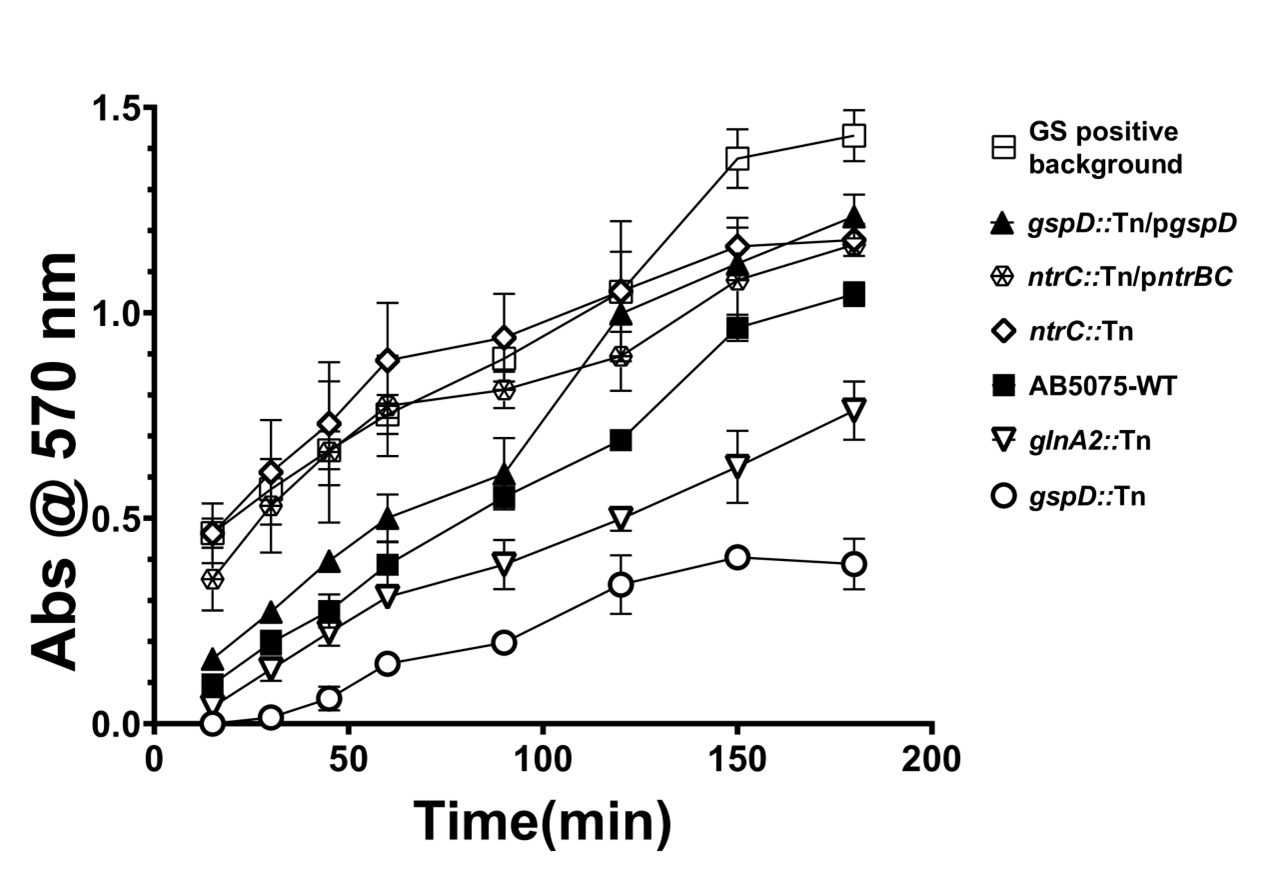

Supplement: S9 Fig — (TIF) [file pone.0341569.s009.tif]

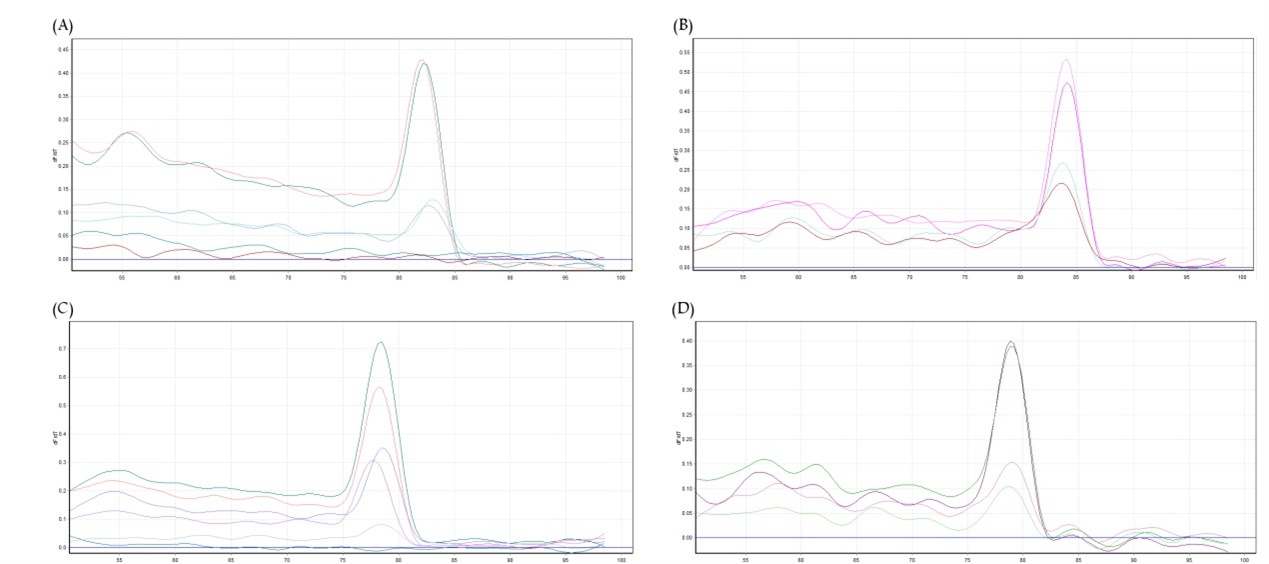

Supplement: S10 Fig — Curves are obtained from real-time PCR reactions demonstrating the specificity and efficiency of primers used for the genes of interest and the 16S rRNA gene. (A) 16S rRNA, (B) glnA1, (C) glnA2, and (D) ntrC. (TIF) [file pone.0341569.s010.tif]

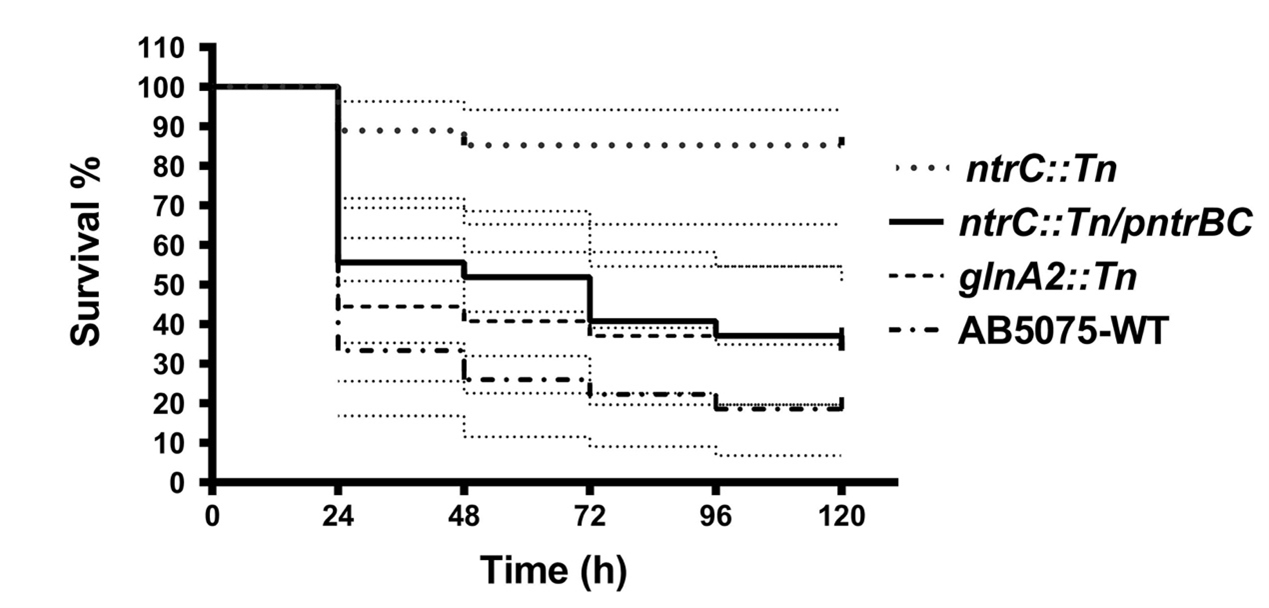

Supplement: S11 Fig — Dotted lines around each curve represent 95% confidence intervals. Survival differences among groups were analyzed and considered statistically significant (p = 0.001). (TIF) [file pone.0341569.s011.tif]
