# Supplementary material for: Transcriptional regulator NtrC modulates nitrogen assimilation, virulence, and the extracellular glutamine synthetase activity in Acinetobacter baumannii
Source: PLoS One. 2026 Jan 23;21(1):e0341569. doi: 10.1371/journal.pone.0341569 (PMC12829801; doi:10.1371/journal.pone.0341569)
Supplement: S1 Table — (DOCX) [file pone.0341569.s012.docx]

**Table. S1** **List of oligonucleotide primers used in this study**

| **Primer** | **Sequence (5′– 3′)** | **Description** |
| --- | --- | --- |
| AJ002 | 5′-AAGGTGGTTACTTCCCAGTTC-3′ | Forward qPCR primer for *ABUW_1207* gene. It is expected to bind to the 5ʹ-end of the open reading frame (ORF) of the *glnA1* gene, starting at nucleotide number 530 on the sense strand. |
| AJ003 | 5′-CTTCGTGGTGGTGTACTTCTAC-3′ | Reverse qPCR primer for the *ABUW_1207* gene. It is expected to bind to the 3ʹ-end of the ORF of the *glnA1* gene, starting at nucleotide number 624 on the antisense strand. |
| AJ011 | 5′-CCGCGACAATTCTGACATCTA-3′ | Forward qPCR primer for *ABUW_1732*. It is expected to bind to the 5ʹ-end of the ORF of the *ntrC* gene, starting at nucleotide number 821 on the sense strand. |
| AJ012 | 5′-CATGCCATTTGAAACCCAGAC-3′ | Reverse qPCR primer for *ABUW_1732*. It is expected to bind to the 3ʹ-end of the ORF of the *ntrC* gene, starting at nucleotide number 735 on the antisense strand. |
| AJ015 | 5′-CAGACGCACAAAATCAACGC-3′ | Forward qPCR primer for *ABUW_1586*. It is expected to bind to the 5ʹ-end of the ORF of the *glnA2* gene, starting at nucleotide number 1212 on the sense strand. |
| AJ016 | 5′-ATGTGCAGGTTTTGGTAGCG-3′ | Reverse qPCR primer for *ABUW_1586*. It is expected to bind to the 3ʹ-end of the ORF of the *glnA2* gene, starting at nucleotide number 1097 on the antisense strand. |
